# Participant Evaluation of a Telephone-Based Osteoarthritis Self-Management Program, 2006-2009

**Published:** 2012-03-22

**Authors:** Nina R. Sperber, Hayden B. Bosworth, Cynthia J. Coffman, Karen A. Juntilla, Jennifer H. Lindquist, Eugene Z. Oddone, Tessa A. Walker, Morris Weinberger, Kelli D. Allen

**Affiliations:** Health Services Research and Development Service, Durham VA Medical Center. Dr Sperber is also affiliated with the Department of Medicine, Duke University Medical Center, Durham, North Carolina; Health Services Research and Development Service, Durham VA Medical Center; Department of Medicine, Duke University Medical Center; and Department of Psychiatry and Behavioral Sciences, School of Nursing, Center for Aging and Human Development, Duke University, Durham, North Carolina; Health Services Research and Development Service, Durham VA Medical Center; and Department of Biostatistics and Bioinformatics, Duke University Medical Center, Durham, North Carolina; 1Health Services Research and Development Service, Durham VA Medical Center, Durham, North Carolina; 1Health Services Research and Development Service, Durham VA Medical Center, Durham, North Carolina; Health Services Research and Development Service, Durham VA Medical Center; Department of Medicine, Duke University Medical Center; and Department of Psychiatry and Behavioral Sciences, School of Nursing, Center for Aging and Human Development, Duke University, Durham, North Carolina; 1Health Services Research and Development Service, Durham VA Medical Center, Durham, North Carolina; Health Services Research and Development Service, Durham VA Medical Center, Durham, North Carolina; and Department of Health Policy and Management, Gillings School of Global Public Health, University of North Carolina, Chapel Hill, North Carolina; Health Services Research and Development Service, Durham VA Medical Center; Department of Medicine, Duke University Medical Center; and Department of Psychiatry and Behavioral Sciences, School of Nursing, Center for Aging and Human Development, Duke University, Durham, North Carolina

## Abstract

**Introduction:**

Self-management support interventions can help improve osteoarthritis outcomes but are underused. Little is known about how participants evaluate the helpfulness of these programs. We describe participants' evaluations of a telephone-based, osteoarthritis self-management support intervention that yielded improved outcomes in a clinical trial.

**Methods:**

Participants were 140 people in the intervention arm of the trial who completed an end-of-trial survey. We used mixed methods to describe participants' perceived helpfulness of the program and its components. We compared ratings of helpfulness according to participant characteristics and analyzed themes from open-ended responses with a constant comparison approach. We calculated Pearson correlation coefficients between perceived helpfulness and changes in pain, function, affect, and self-efficacy.

**Results:**

The average rating of overall helpfulness on a scale from 1 to 10 was 7.6 (standard deviation, 2.3), and more than 80% of participants agreed that each component (phone calls, educational material, setting goals and action plans) was helpful. Participants had better perceived helpfulness ratings than their counterparts if they were nonwhite, had limited health literacy, had no college education, had perceived inadequate income, were older, had a spouse or were living together in a committed relationship, and had greater symptom duration and less pain. Ratings of helpfulness increased with greater improvement in outcomes. Participants frequently mentioned the health educator's calls as being helpful for staying on task with self-management behaviors.

**Conclusion:**

Participants viewed this intervention and each of its components as helpful for improving osteoarthritis symptoms. In addition to the improvements in objective outcomes seen in the clinical trial, these results provide further support for the dissemination of self-management support interventions.

## Introduction

Self-management is an essential but underused tool for addressing arthritis, which is expected to affect 67 million American adults (25% of the projected US adult population) by 2030 (1,2). Self-management support interventions help people work toward meaningful goals regarding the medical, behavioral, and emotional aspects of their disease (3). Arthritis self-management programs can help improve pain, function, and other outcomes of patients with osteoarthritis, the most common arthritic condition; however, little is known about how patients perceive the helpfulness of these programs (4). Eliciting patients' experiences can help determine if there is concordance between outcomes that are clinically and personally important; this information can enhance evidence-based interventions so that they are well-matched to patients' needs (4-6). Our objective was to describe participant evaluations of a telephone-based self-management support intervention for people with osteoarthritis that yielded modest improvements in pain and some aspects of physical function in a clinical trial (7).

## Methods

### Overview

This study is a secondary analysis from a 12-month clinical trial of an osteoarthritis self-management support intervention conducted at the Durham Veterans Affairs Medical Center (VAMC) (7,8) between 2006 and 2009. The secondary analysis was restricted to intervention-arm participants who completed the end-of-trial evaluation survey (140 of 172 participants, 81% participation). We describe their ratings of the helpfulness of the program (collected at the end of the trial), comparisons according to participants' characteristics, and relationship of ratings with change in objective outcomes.

### Participants and procedure

Inclusion criteria for the clinical trial were enrollment in primary care at the VAMC; a physician diagnosis of hip or knee osteoarthritis; and persistent, current joint symptoms. Exclusion criteria were having psychoses, dementia, other health conditions that would likely prevent participation in the study, or other rheumatological conditions; being on a waiting list for arthroplasty; and participation in another osteoarthritis-related or lifestyle intervention study. Each participant received written and audio versions of osteoarthritis self-management educational materials, consisting of 10 modules: 1) the basics of osteoarthritis and self management, 2) exercise, 3) healthy eating and weight management, 4) medications, 5) joint injections and surgery, 6) talking with your doctor, 7) joint care, 8) complementary and alternative therapies, 9) stress management, and 10) sleep. Participants received monthly phone calls from a health educator to review key points from the modules, develop weekly self-management goals and action plans, and engage in problem solving. Participants chose the order of topics after covering the basic information module. This study was reviewed and approved by the institutional review board of the VAMC.

### Measures

**Evaluation survey**

The survey was part of the end-of-trial follow-up assessment for participants in the intervention arm of the clinical trial and was administered in English either in-person (n = 112) or over the telephone (n = 28). Participants received $10 for completing follow-up assessments (7). Perceived helpfulness of the program was assessed by asking participants to rate on a scale from 1 ("not at all helpful") to 10 ("very helpful"). Participants were also asked whether specific components (health educator's calls, written or audio educational materials, and goal setting and developing action plans) helped them improve their osteoarthritis symptoms. These items were measured using a 5-point Likert scale (1 = strongly agree to 5 = strongly disagree). Likelihood of participation was assessed by the question, "If the VA offered an arthritis self-management course like this one at no cost to you, would you participate?" Possible responses were yes, no, or maybe. Participants were then asked, "If the VA offered an arthritis self-management course like this one for a fee, would you pay? How much would you pay to participate?" Possible responses were 0/would not participate for a fee, $5 to $19, $20 to $29, $30 to $39, and $40 or more. Participants were also asked an open-ended question: "What part(s) of the arthritis self-management program were most helpful to you?"

**Participant characteristics**

We assessed the following characteristics at baseline: age (≤54 y, 55-64 y, ≥65 y); race (white, nonwhite); education (at least some college, no college); health literacy, assessed using the Rapid Estimate of Adult Literacy in Medicine (REALM) (high school, eighth grade and below) (9); self-reported perceived inadequate income (assessed with agreement or disagreement with the statements, "You have money to pay the bills, but only because you have to cut back on things" and "You are having difficulty paying the bills, no matter what you do"); marital status (married or living together in a committed relationship or not); self-reported years experiencing arthritis symptoms (quartiles: 1-6, 7-13, 14-20, 21-64); and self-reported general health (excellent, very good, or good vs fair or poor).

**Calls completed**

We also examined the number of completed monthly calls and dichotomized responses as 1 to 8 or 9 to 12. (New information was delivered for the first 9 calls, and remaining calls were reserved for review or participant questions; therefore, participants who completed at least 9 calls received all intervention content.)

**Osteoarthritis outcomes**

Outcomes were scores from pain, mobility, and affect subscales of the Arthritis Impact Measurement Scales (AIMS2) (10); a pain visual analogue scale (VAS) (11); and the Arthritis Self-Efficacy Scale (12), which were collected at baseline and the end of the trial. The AIMS2 pain subscale consists of 5 items assessing typical pain, pain severity, and pain during specific times of the day. The AIMS2 mobility subscale consists of 5 items that ask about one's ability to get around outside of the home. The AIMS2 affect subscale consists of 10 items that address mood and tension. All items on these subscales are measured on a 5-point Likert scale ("all days" to "no days"); scores range from 0 to 10, and higher scores indicate worse outcomes. The pain VAS is a 10-cm line on which participants mark their average pain during the past 2 weeks, using anchors of "no pain" and "pain as bad as it can be." The Arthritis Self-Efficacy Scale measures how certain patients are that they can perform 8 activities or tasks related to arthritis. Items are scored on a Likert scale (1 = very uncertain to 10 = very certain); scores range from 1 to10, and higher scores indicate better self-efficacy.

### Data analysis

We used a quantitatively driven mixed-method design in which we separately analyzed open-ended responses to complement quantitative findings (13). We created contingency tables for each closed-ended question about program helpfulness to describe responses for the total sample and by participant characteristics, baseline pain VAS score (dichotomized: ≤5 = low pain, >5 = high pain [14,15]), and completed calls. For all closed-ended questions, we combined the *strongly agree* and *agree* response categories (vs *neither agree nor disagree,* *don't know*, *disagree,* and *strongly disagree*). We calculated Pearson correlation coefficients to examine associations between perceived helpfulness of the program and change in each osteoarthritis outcome from baseline to follow-up. One researcher coded qualitative responses with a priori (calls, educational materials, goal setting) and emergent codes (16) and continuously compared the codes to arrive at conceptually distinct categories (17). Because there is no established definition of a clinically meaningful difference in perceptions of helpfulness and value, we commented on differences close to 0.5 points or more for overall helpfulness rating and at least 5% across categories for the categorical variables. We used SAS version 9.2 (SAS Institute, Inc, Cary, North Carolina) and ATLAS.ti version 6.1 (ATLAS.ti Scientific Software Development GmbH, Berlin, Germany) software.

## Results

The mean age of this sample was 60 years (Table 1). Most participants were male, and approximately half were white. On a scale from 1 to 10, participants' mean rating of the program's helpfulness was 7.6 (Table 2). More than 80% of participants overall strongly agreed or agreed that each component helped improve their osteoarthritis symptoms. Eighty-five percent said they would participate in this program if the VA offered it to them at no cost. When asked about paying to participate, 36% said that they would not participate for a fee, 34% said that they would pay $1 to $29, and 30% said that they would be willing to pay $30 or more.

Of the 140 participants who responded to the survey, 31 (22%) completed 1 to 8 calls and 109 (78%) completed 9 to 12 calls (Table 3). Mean ratings of perceived overall program helpfulness by participant characteristics ranged from 7.0 to 8.1. The rating of overall helpfulness increased with age. Participants who were nonwhite, had no college education, had a health literacy level of eighth grade or below, had perceived inadequate income, reported less pain, and were married or living together in a committed relationship reported higher mean levels of perceived helpfulness than their counterparts. Participants with the longest self-reported duration of osteoarthritis symptoms (21-64 y) had the highest average rating of overall helpfulness. Participants who completed 9 to 12 calls rated the overall helpfulness on average as 7.8, and participants who completed 1 to 8 calls had an average score of 7.0.

More than 68% of participants across the different characteristics evaluated each of the 3 intervention components as being helpful (*agree* or *strongly agree*) (Table 3). Participants who were older, nonwhite, lacked college education, had a low health literacy level, were married or living together in a committed relationship, had greater self-reported duration of osteoarthritis symptoms, or who reported less pain were more likely than their counterparts to agree that the health educator's calls were helpful. Participants who were older, nonwhite, had a low health literacy level, were married or living together in a committed relationship, or had less pain were more likely to agree that the educational materials were helpful. Participants who had the longest self-reported duration of osteoarthritis symptoms (14-64 y) were more likely to rate the educational materials as helpful than participants who reported a shorter duration of symptoms. Participants who were older, had a low health literacy level, did not report perceived inadequate income, were married or living together in a committed relationship, reported 1 to 20 years of osteoarthritis symptoms, had better self-reported general health, and had less pain were more likely than their counterparts to agree that setting goals and action plans were helpful. Participants who completed 9 to 12 calls rated the program components as helpful more (84%-90%) than participants who completed only 1 to 8 calls (68%-81%).

Correlations of perceived program helpfulness with changes in the pain VAS and AIMS2 subscale scores were negative (*r* = −0.10 to −0.17), indicating that as symptom levels got worse (higher scores at follow-up than at baseline), perceived helpfulness ratings were worse, or that as symptom levels improved (lower scores at follow-up than at baseline), perceived helpfulness ratings were better (Table 4). There was a positive correlation of perceived program helpfulness with arthritis self-efficacy (*r* = 0.17), indicating that perceived helpfulness ratings and self-efficacy increased (higher scores at follow-up) and decreased together.

When asked which part or parts of the program were most helpful, participants most frequently mentioned the health educator's calls (44 of 140, 31%), followed by educational materials (written and audio) (20 of 140, 14%) and goal setting (11 of 140, 8%). Participants also commonly said that it was helpful to learn about exercise (42 of 140, 30%) and healthy eating and weight management (20 of 140, 14%) for managing their osteoarthritis symptoms.

### Health educator's calls

Of those who mentioned the calls as being the most helpful component of the intervention, almost half (21 of 44, 48%) said that the health educator's contact enabled them to stay on task with the educational materials and goal setting. One person said, "The monthly calls helped me stay aware of doing something rather than just trying to live with my arthritis." Several participants (8 of 44, 18%) found it encouraging to discuss their osteoarthritis with someone who understood their situation. As a participant stated, "[It was] emotionally and mentally satisfying to talk with the health educator, because I had some fears regarding my arthritis." Some (6 of 44, 14%) also said that the calls provided an educational benefit by imparting and clarifying information related to the modules.

### Educational materials

Forty percent (8 of 20) of those who mentioned educational materials said that the information helped them understand more about their osteoarthritis and how to better manage it. One participant said, "The audio cassette explained things I did not realize about osteoarthritis, such as the causes, prevention, and why [and] how it affected me." Another participant said, "It gave me more knowledge about my options for arthritis. It's hard to do anything if you don't know how to do it." Some (4 of 20, 20%) described the written materials as an easy-to-read reference and said that the materials were helpful combined with calls. As a participant said, "I liked the book with the short chapters, making it easy to read and understand, and [the health educator] reinforced it when she called." Two participants specifically said the information was helpful for their pain management.

### Goal setting

Of participants who said that goals were most helpful, some (5 of 11, 45%) indicated that the consistent calls helped them adhere to their goals, and several (3 of 11, 27%) said that goal setting spurred them to take an active role in managing their symptoms. One participant said, "Speaking to the educator on a monthly basis . . . gave me the incentive to go on for the next month." Another participant said, "Setting the goals . . . made me realize there are things I can do to help myself with the pain. It helped my mental ability to deal with the arthritis."

### Exercise and healthy eating/weight management

Some participants who mentioned exercise (7 of 42, 17%) or healthy eating and weight management (2 of 20, 10%) said that implementing these behaviors helped with controlling their pain levels. However, 1 participant stated that "The exercise helped increase my strength, even improving the ability to stand up, but not with diminishing my pain level. I have more endurance to be able to walk a distance, but I still hurt a lot when I return to the house."

## Discussion

This study is one of the first to describe how participants view the helpfulness of an osteoarthritis self-management support intervention for improving their symptoms (4). Comparing participants' evaluations with clinical trial outcomes can help indicate the extent to which personal experiences align with traditional objective outcomes. Overall, our results suggest that participants viewed the intervention as beneficial.

Perceived helpfulness varied by socioeconomic characteristics. In general, participants with lower health literacy, who lacked college education, or who had perceived inadequate income were more likely than their counterparts to find 1 or more aspects of the program helpful. This pattern suggests that people with limited resources may need more information about the nature and management of their disease (18). Although responses to open-ended questions were not examined according to participant characteristics, participants commonly expressed appreciation for the information that they received on how to improve their experience with osteoarthritis, as well as the easy-to-understand and multimodal delivery of the program. These results highlight the importance of making self-management support interventions appropriate and accessible to people with lower education and health literacy levels, particularly because these patients are at greater risk for more severe osteoarthritis symptoms (1).

We found that higher proportions of nonwhites than whites reported that the health educator's calls and educational materials were helpful. This difference could partially be explained by the higher numbers of nonwhites with limited health literacy or perceived inadequate income and fewer numbers with at least some college education in our sample, all of which were also associated with greater agreement that the overall program or individual components were helpful. Other researchers have also found that racial disparities in health status and osteoarthritis outcomes are explained by socioeconomic variables (18-20). However, other cultural, psychosocial, or clinical characteristics may have contributed to these racial differences in program helpfulness.

Participants who were married or living in a committed relationship had higher ratings of program helpfulness than participants who were not. Prior research has shown that close relationships are important for chronic disease outcomes in general (21), but to our knowledge this is the first study to examine perceptions of helpfulness of a self-management program according to relationship status. Participants living in close relationships may have had more support to carry out their goals and action plans during the intervention period.

Both older age and more years with osteoarthritis symptoms were associated with higher mean ratings of overall program helpfulness, and, in particular, perceived helpfulness of the educational materials and the health educator's calls. People who faced more age-related limitations or symptom persistence may have had a greater need for this type of program and, therefore, responded more strongly to the emotional and informational supports.

Participants who reported less pain were more likely than those who reported a high level of pain to find program components helpful. Patients with more pain may need a more intense behavioral program, greater coordination with clinical care, or additional treatments (eg, knee braces, joint injections, joint replacement) to perceive substantial changes in symptoms. However, although people may not perceive substantial benefits related to pain from these types of programs, other clinically important outcomes, including mental health, physical function, and acceptance of limitations, can be influenced (22,23).

Patient perceptions of program helpfulness were high, although changes in outcomes such as pain and function were moderate (7). These findings indicate that the intervention may have affected patients in ways that are not captured completely by traditional outcome measures. Because this program was designed to enhance osteoarthritis self-efficacy, patients' notions of helpfulness may have reflected a feeling of being more in command of their osteoarthritis, as has been previously reported (4). The intervention did result in a greater increase in self-efficacy, compared with usual care and the health education group (7). The monthly health educator's calls may be a source of this effect for many participants, as reflected in responses to open-ended questions, by providing consistent encouragement to help them stay on task with their osteoarthritis self-management goals and reinforcement to help them better grasp the informational material.

This study has limitations. Because this study was conducted at 1 VA medical center and consisted of a primarily male sample, generalizability may be limited. Additionally, patients may have inflated their subjective responses when talking to a study team member. We tried to minimize this potential source of bias by not having the health educator who delivered the intervention conduct these interviews.

These results provide support for ongoing efforts to increase dissemination of osteoarthritis self-management support interventions. These programs are viewed as being even more beneficial by some patient subgroups, including racial minorities and those with lower socioeconomic status, who are also at greater risk for worse osteoarthritis outcomes and who may benefit most from targeted osteoarthritis self-management support interventions.

## Figures and Tables

**Table 1.** Baseline Characteristics of Participants (n = 140) in the Veterans Affairs Medical Center Osteoarthritis Self-Management Support Intervention, Durham, North Carolina, 2006-2009

**Variable**	Value^a^
**Age, mean (SD), y**	59.8 (10.3)
**Male sex**	126 (90)
**Race**	
White	75 (54)
Black/African American	62 (44)
Other	3 (2)
**Body mass index, kg/m^2^**	
≥30.0 (Obese)	82 (58.6)
25.0-29.9 (Overweight)	47 (35)
18.5-24.9 (Normal weight)	9 (6.4)
<18.5 (Underweight)	2 (1)
**At least some college**	94 (67)
**Health literacy^b^**	
High school	92 (66)
8th grade and below	45 (32)
**Self-reported perceived inadequate income^c^**	41 (29)
**Married or living together in a committed relationship**	102 (73)
**Self-reported years with arthritis symptoms, y, mean (SD)**	17.4 (13.2)
**Excellent, very good, or good self-reported general health**	98 (70)
**Pain VAS baseline score,^d^ mean (SD)**	5.8 (2.3)
**AIMS2^e^ pain baseline score, mean (SD)**	6.0 (2.3)
**AIMS2 mobility baseline score, mean (SD)**	1.7 (2.0)
**AIMS2 mood baseline score, mean (SD)**	2.7 (2.1)
**AIMS2 tension baseline score, mean (SD)**	4.9 (2.7)
**Arthritis self-efficacy baseline score,^f^ mean (SD)**	5.6 (2.0)

Abbreviations: SD, standard deviation; VAS, Visual Analog Scale; AIMS2, Arthritis Impact Measurement Scales.

a Values are expressed as n (%) unless otherwise indicated.

b Assessed using the Rapid Estimate of Adult Literacy in Medicine (REALM).

c "You have money to pay the bills, but only because you have to cut back on things," or "You are having difficulty paying the bills, no matter what you do."

d The VAS is measured on a scale of 1 to 10, with 1 being "no pain" and 10 being "pain as bad as it can be."

e The potential range of the AIMS2 measures is 0-10, with lower scores indicating better health status.

f The potential range of arthritis self-efficacy is 0-10, with higher scores indicating better self-efficacy.

**Table 2.** Participant (n = 140) Evaluation Questions and Responses from the Veterans Affairs Medical Center Osteoarthritis Self-Management Support Intervention, Durham, North Carolina, 2006-2009

**Question**	Value^a^
**On a scale of 1-10, with 1 being not at all helpful and 10 being very helpful, how helpful was this program for you? (mean [SD])**	7.6 (2.3)
**The health educator's calls helped me improve my arthritis symptoms.^b^**	113 (81)^c^
**The educational material (written or audio) helped me improve my arthritis symptoms.^b^**	119 (85)^c^
**Setting goals and action plans helped me improve my arthritis symptoms.^b^**	121 (86)^c^
**If the VA offered an arthritis self-management course like this one at no cost to you, would you participate?**	119 (85)^d^
**If the VA offered an arthritis self-management course like this one for a fee, would you pay? How much would you pay to participate? (Expressed as $)**	
0/Would not participate for a fee	51 (36)
1-4	7 (5)
5-19	21 (15)
20-29	19 (14)
30-39	3 (2)
≥40	39 (28)

Abbreviations: SD, standard deviation; VA, Veterans Affairs.

a Values are expressed as n (%), unless otherwise indicated.

b The 5-point scale ranged from 1 (strongly agree), to 3 (neither agree nor disagree), to 5 (strongly disagree).

c Number and percentage reflect combined "strongly agree" and "agree" categories.

d Number and percentage reflect participants who answered yes.

**Table 3.** Perceived Helpfulness of the Veterans Affairs Medical Center Osteoarthritis Self-Management Support Intervention, by Participant Characteristics, Durham, North Carolina, 2006-2009

Baseline Participant Characteristic	Question				

	How helpful was this program for you?^b^		Health educator's calls helped me improve my arthritis symptoms^c^^,^ d	Educational material (written and audio) helped me improve my arthritis symptoms^c^^,^ d	Setting goals and action plans helped me improve my arthritis symptoms^c^^,^ d

	N^a^	Mean (SD)	n (%)	n (%)	n (%)
**Age, y**					
≤54	37	7.2 (2.5)	28 (76)	29 (78)	29 (78)
55-64	68	7.6 (2.3)	57 (84)	59 (87)	62 (91)
≥65	35	8.1 (2.0)	28 (80)	31 (89)	30 (85)
**Race**					
White	75	7.5 (2.4)	57 (76)	61 (81)	65 (87)
Nonwhite	65	7.7 (2.1)	56 (86)	58 (89)	56 (86)
**Education**					
At least some college	94	7.5 (2.3)	73 (78)	81 (86)	81 (86)
No college	46	7.9 (2.1)	40 (87)	38 (83)	40 (87)
**REALM**					
High school	92	7.5 (2.3)	70 (76)	76 (83)	78 (85)
8^th^ grade or below	45	8.0 (2.1)	42 (93)	40 (89)	41 (91)
**Self-reported perceived inadequate income**					
No	98	7.5 (2.2)	79 (81)	82 (84)	86 (88)
Yes	41	7.9 (2.3)	33 (81)	36 (88)	34 (83)
**Married or living together in a committed relationship**					
No	38	7.1 (2.8)	26 (68)	29 (76)	29 (76)
Yes	102	7.8 (2.0)	87 (85)	90 (88)	92 (90)
**Self-reported years with arthritis symptoms**					
1-6	31	7.5 (2.3)	23 (74)	26 (84)	29 (94)
7-13	34	7.4 (2.2)	26 (76)	27 (79)	28 (82)
14-20	38	7.4 (2.4)	31 (82)	33 (87)	35 (92)
21-64	37	8.1 (2.1)	33 (89)	33 (89)	29 (78)
**Excellent, very good, or good self-reported general health**					
Yes	98	7.7 (2.2)	78 (80)	83 (85)	86 (88)
No	42	7.5 (2.4)	35 (83)	36 (86)	35 (83)
**Pain VAS score**					
0-5	54	7.5 (2.2)	47 (87)	50 (93)	50 (93)
>5	86	7.7 (2.3)	66 (77)	69 (80)	71 (83)
**Completed calls**					
1-8	31	7.0 (2.3)	21 (68)	25 (81)	23 (74)
9-12	109	7.8 (2.2)	92 (84)	94 (86)	98 (90)

Abbreviations: REALM, Rapid Evaluation of Adult Literacy in Medicine; VAS, visual analog scale.

a Values for N may not sum to 140 because of missing data.

b Measured on a scale of 1 = not at all helpful to 10 = very helpful.

c Original 5-point scale ranged from 1 (strongly agree), to 3 (neither agree nor disagree), to 5 (strongly disagree).

d Counts and percentages reflect combined "strongly agree" and "agree" categories.

**Table 4.** Correlations of Change in Osteorthritis Outcomes (Follow-Up to Baseline) With Perceived Helpfulness of the Veterans Affairs Medical Center Osteoarthritis Self-Management Support Intervention, Durham, North Carolina, 2006-2009

**Arthritis Outcome**	Mean Change (SD)	Correlation With Perceived Program Helpfulness, *r* a
**Pain VAS score^b^**	−1.04 (2.2)	−0.11
**AIMS2 pain score^b^**	−0.85 (2.2)	−0.15
**AIMS2 mobility score^b^**	−0.31 (1.6)	−0.13
**AIMS2 affect score**		
AIMS2 mood score^b^	−0.18 (1.7)	−0.17
AIMS2 tension score^b^	−0.30 (2.2)	−0.10
**Arthritis self-efficacy score^c^**	0.53 (1.9)	0.17

Abbreviations: SD, standard deviation; VAS, Visual Analog Scale; AIMS2, Arthritis Impact Measurement Scales.

a Assessed by the question, "On a scale of 1 to 10, with 1 being not helpful at all and 10 being very helpful, how helpful was this program for you?"

b A negative correlation indicates that as symptom levels (pain, mobility, affect) got worse (higher scores at follow-up than baseline), perceived helpfulness ratings were worse, or as symptom levels improved (lower scores at follow-up), perceived helpfulness ratings were better.

c A positive correlation indicates that perceived helpfulness ratings and self-efficacy increased and decreased together.
